# Extinction risks of a Mediterranean neo-endemism complex of mountain vipers triggered by climate change

**DOI:** 10.1038/s41598-019-42792-9

**Published:** 2019-04-19

**Authors:** Mohsen Ahmadi, Mahmoud-Reza Hemami, Mohammad Kaboli, Mansoureh Malekian, Niklaus E. Zimmermann

**Affiliations:** 1 Department of Natural Resources, Isfahan University of Technology, Isfahan, 8415683111, Iran; 2 Department of Environmental Sciences, Faculty of Natural Resources, University of Tehran, Karaj, Iran; 3 Swiss Federal Research Institute WSL, CH-8903 Birmensdorf, Switzerland

**Keywords:** Climate-change ecology, Biodiversity

## Abstract

Climate change is among the most important drivers of biodiversity decline through shift or shrinkage in suitable habitat of species. Mountain vipers of the genus *Montivipera* are under extreme risk from climate changes given their evolutionary history and geographic distribution. In this study, we divided all *Montivipera* species into three phylogenetic-geographic *Montivipera* clades (PGMC; Bornmuelleri, Raddei and Xanthina) and applied an ensemble ecological niche modelling (ENM) approach under different climatic scenarios to assess changes in projected suitable habitats of these species. Based on the predicted range losses, we assessed the projected extinction risk of the species relative to IUCN Red List Criteria. Our result revealed a strong decline in suitable habitats for all PGMCs (63.8%, 79.3% and 96.8% for Xanthina, Raddei and Bornmuelleri, respectively, by 2070 and under 8.5 RCP scenario) with patterns of altitudinal range shifts in response to projected climate change. We found that the mountains close to the Mediterranean Sea are exposed to the highest threats in the future (84.6 ± 9.1 percent range loss). We also revealed that disjunct populations of *Montivipera* will be additionally highly isolated and fragmented in the future. We argue that leveraging climate niche projections into the risk assessment provides the opportunity to implement IUCN criteria and better assess forthcoming extinction risks of species.

## Introduction

In recent decades, risks of population declines and local extinctions in many species of reptiles and amphibians have been reported from across the globe^1^. A multitude of factors has been identified as potential main drivers behind these declines, including climate warming and climate change as a widely approved factor^2,3^. However, determining the role of climate change on biodiversity decline is challenging as numerous factors are involved and climate changes vary depending on spatial scales^4^. Conservationists have assessed the biological risks from projected impacts of climate changes using empirical species distribution models^2,5–7^, demographic simulations^8–10^ or phylogenetic proxies^11–14^.

One of the most obvious effects of climate change on biodiversity is the projected change in species geographical ranges^15,16^. The geographic distribution of any species, with some degrees of disequilibrium, is a spatial representation of its ‘*ecological niche*’. Extrinsic factors such as abiotic conditions and species interactions, and intrinsic ones such as dispersal capability and genetic plasticity determine form, dynamic, and evolution of the niche^17^. The interactions between these factors through past evolutionary time-scales determine the current distribution range of the species^18^. In the Anthropocene, the rapid rate of climate change has caused signatures of range loss and range shift in many species, with an observed time lag of population dynamic responses relative to climatic trends^8^. Over the past century, increasing evidence of climate change impacts on biodiversity patterns has been documenting a globally consistent rate of poleward or upward shifts in many species’ geographic distributions^6,7,12,15^, although some species show deviating responses^19,20^.

The consequences of climate change on conservation status and extinction risks have been assessed for a wide array of taxonomic groups. For example, Thomas, *et al*.^21^ estimated that 18–35% of the analyzed species are at risk of losing significant suitable habitat by 2050. A review on ecological and evolutionary responses of biodiversity to recent changes in climate has concluded that range-restricted species (e.g. polar and mountain-dwelling species) are the first group to respond with highest observed extinction rates in response to climate change^22^. Foden, *et al*.^23^ argued that up to 83% of birds, 66% of amphibians and 70% of corals are not currently classified threatened in the Red List despite being projected to be highly sensitive to the impacts of climate change.

Generally, the IUCN assessment of the Red List status relies on published data and on expert knowledge about distribution, population status and ecology of species. A trait-based assessment, which aimed at identifying the important species characteristics associated with high risk of extinction due to climate change^10^, has pointed to the ‘*occupied area*’ (total area of all occupied patches) as being the most sensitive characteristic. The terms ‘*extent of occurrence*’ (EOO) and ‘*area of occupancy*’ (AOO) are two metrics representative of a species’ geographic range (i.e. occupied areas), and are used to assess extinction risks and the Red List status of species (criteria B in the IUCN Red List). Moreover, as the IUCN recommends that either the known, inferred or projected sites of occurrence could be used to estimate the species range^24^, predicting suitable range for the species based on their ecological requirements through an ecological niche modelling (ENM) procedure is a highly suitable tool for conservation assessments. Consequently, ENM (also habitat suitability or species distribution modelling) has widely been used to estimate EOO and AOO, considering the total number and extent of grid cells predicted to provide suitable habitat for a target species^25–27^.

Nevertheless, extinction risk assessments often suffer from uncertainties that arise from different parametrization and model projections^28,29^, species-specific characteristics e.g. dispersal scenarios;^10,30^ and/or taxonomic bias of unresolved or inconsistent phylogenetic relationships^31,32^. Furthermore, issues emerge from insufficient availability of species distribution data^33,34^, and from insufficient scale and resolution of range maps^35,36^, particularly for unknown or data-deficient species. In this situation, projections of AOO and EOO based on spatially explicit models can provide inferred sites of species occurrences that are less affected by sampling efforts and scale issues^27^.

One taxonomic group that is specifically threatened by climate change is Mountain viper species of the recently recognized genus, *Montivipera*^37^ which is an intriguing model of neo-endemism in Mediterranean ecosystems. The interaction of geomorphologic, tectonic and climatic drivers have formed a complex and heterogeneous assemblage in this bioregion^38–40^. The high magnitude of Tertiary-Quaternary climate fluctuations has caused many taxa to undergo repetitive patterns of expansion, contraction and isolation^41,42^. In addition, severe ecological conditions and high spatial divergence in most parts of these Mediterranean ecosystems have resulted in the emergence of a substantial degree of recent speciation events^38^, i.e. neo-endemism^43,44^. As a consequence, the Near and Middle East embrace the richest herpetofauna and harbor the highest diversity of true vipers within the Palearctic bioregion^44,45^.

**Figure 1:**
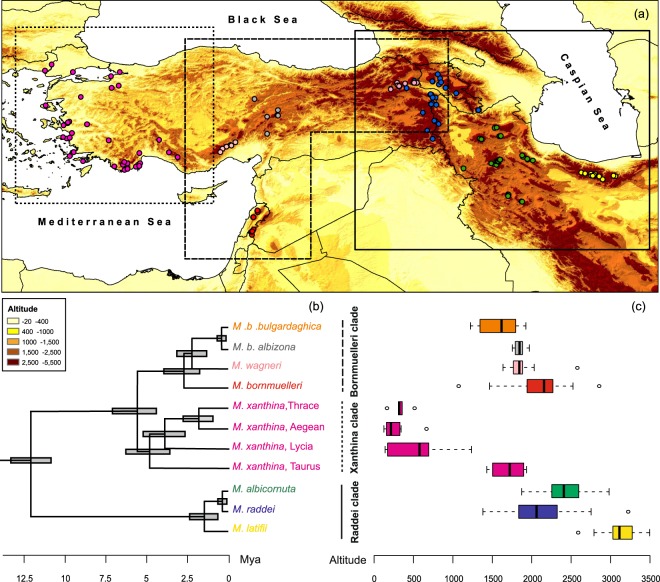
Global distribution of phylogeographic *Montivipera* clades (PGMCs) (**a**), their phylogenetic relationship based on Stümpel, *et al*.^45^ (**b**), and altitudinal distribution patterns in the study area (**c**).

The distribution of *Montivipera* species is constrained by the Mediterranean climate regime from the flat coast of western Turkey to the mountainous landscapes of the Irano-Anatoly and the southern Caucasus regions (Fig. 1). Strong isolations through repeated retractions to isolated mountain tops during late Tertiary climatic fluctuations have caused vicariant allopatric speciation and a rapid rate of diversifications in this genus^7,45^. Generally (and from a phylogenetic viewpoint), *Montivipera* species are categorized into two complexes; Xanthina and Raddei^37^, yet their taxonomy remains debated^45^. The Xanthina complex includes the species *M. xanthina*, *M. wagneri, and M. bornmuelleri* with two species-subspecies *M. b. albizona* and *M. b. bulgardaghica*. The Raddei complex contains *M. raddei*, *M. latifii*, *M. albicornuta* and *M. kuhrangica*. Isolated distribution ranges and population declines due to habitat loss and illegal collection for antivenin production, especially of the Raddei complex^46^, have resulted in all *Montivipera* species being classified as threatened in the Red List of the IUCN, except for *M. xanthina*.

The restricted current distribution ranges and small population sizes of all *Montivipera* species, and the ongoing threats from climate change urge for an assessment of their vulnerability and for developing conservation priorities in response to projected climate trends. Here, we assess the risks of mountain vipers to lose habitat from climate change in a hotspot of the Palearctic herpetofauna. More specifically, we address the following questions: (1) how could projected climate change affect the area of suitable habitat (AOO) in mountain vipers; (2) to what degree could projected climate change affect patch connectivity and isolation; and (3) how do these changes affect the IUCN risk assessment criteria of mountain vipers. To answer these questions, we used ENM to derive climatically suitable habitats as an index of currently occupied areas (AOO) of *Montivipera* genus in the eastern Mediterranean climatic regime (Irano-Anatoly and the southern Caucasus). We then removed anthropogenically converted land areas from the modelled habitats and measured projected shifts and/or shrinkages of the AOO to assess current and forthcoming extinction risks of the species under future climates. By using landscape ecology analyses, we further assessed the projected long-term persistence of the species complexes through tracking the spatial configuration of suitable habitats.

## Results

**Figure 2:**
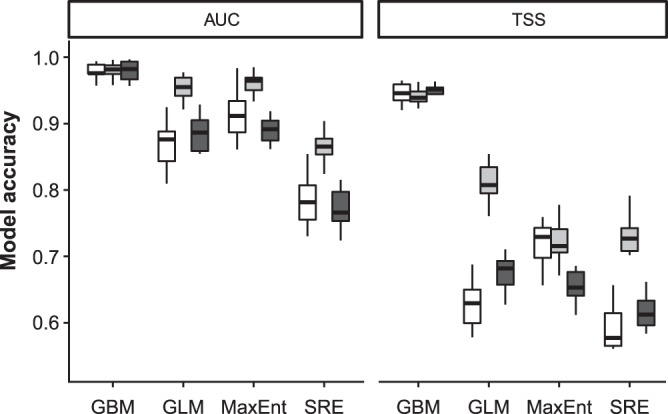
Values of the model’s accuracy based on AUC and TSS of the 10-replicated models for Bornmuelleri (white), Raddei (light gray) and Xanthina (dark grey) clades. GBM; generalized boosted model, GLM; generalized linear model, MaxEnt; maximum entropy, SRE; surface range envelop.

**Figure 3:**
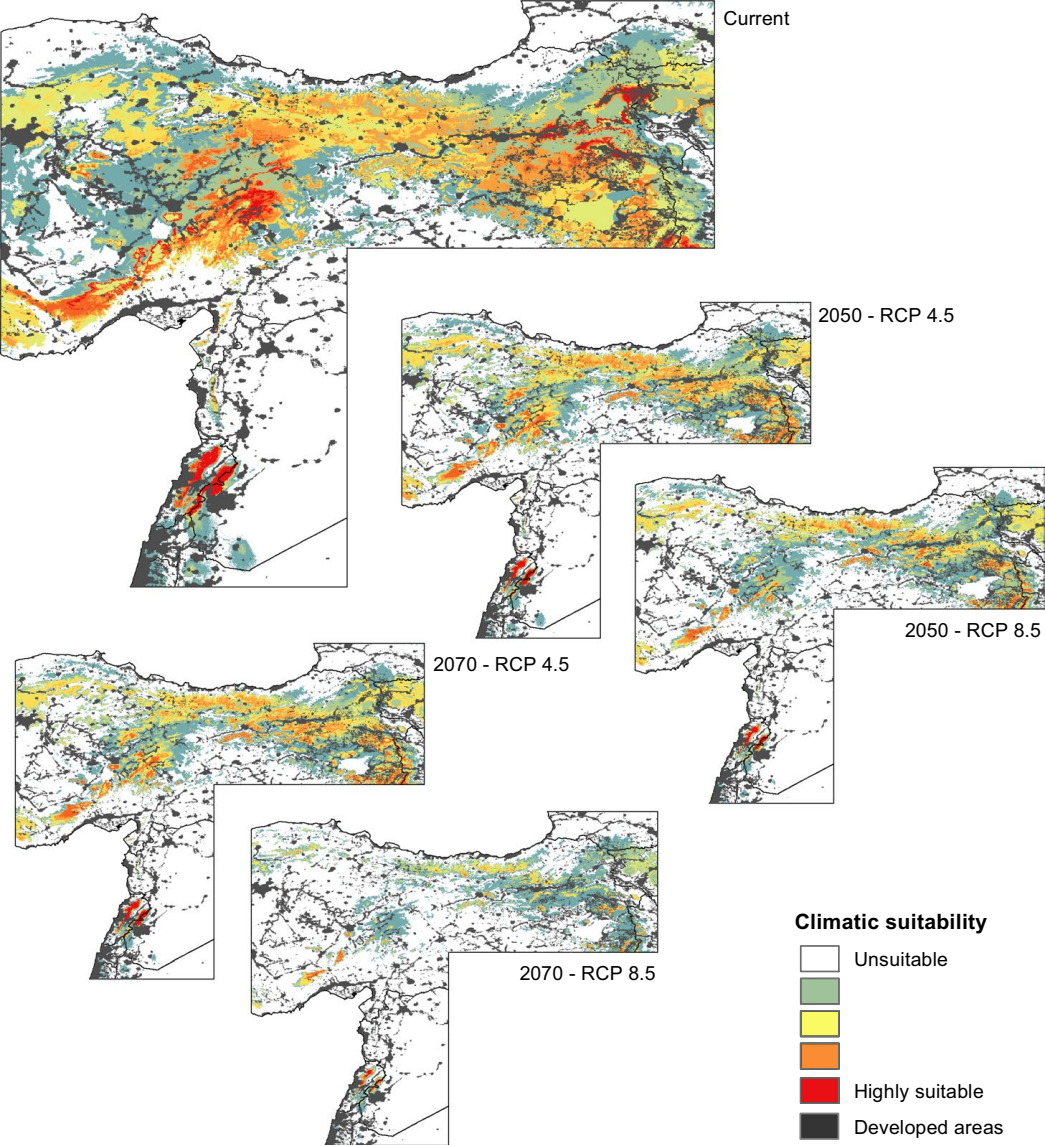
Predicted climatically suitable areas for the Bornmuelleri clade for current and future, 2050 and 2070, with two RCP scenarios. Suitability maps are average calculations of the four GCM models for each time period and RCP scenario. Grey colors show areas with human footprint greater than 50.

**Figure 4:**
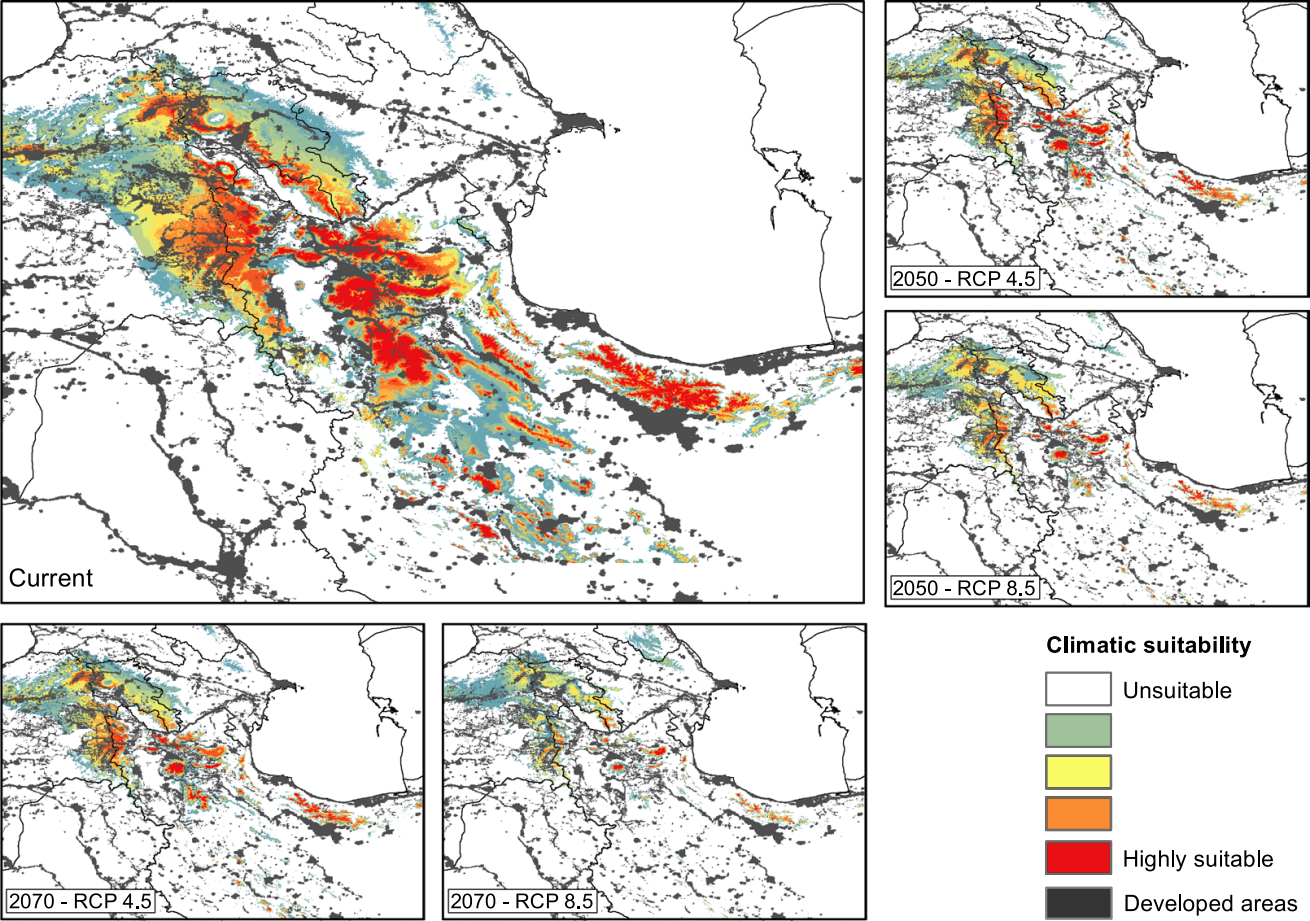
Predicted climatically suitable areas for the Raddei clade for current and future, 2050 and 2070, with two RCP scenarios. Suitability maps are average calculations of the four GCM models for each time period and RCP scenario. Grey colors show areas with human footprint greater than 50.

**Figure 5:**
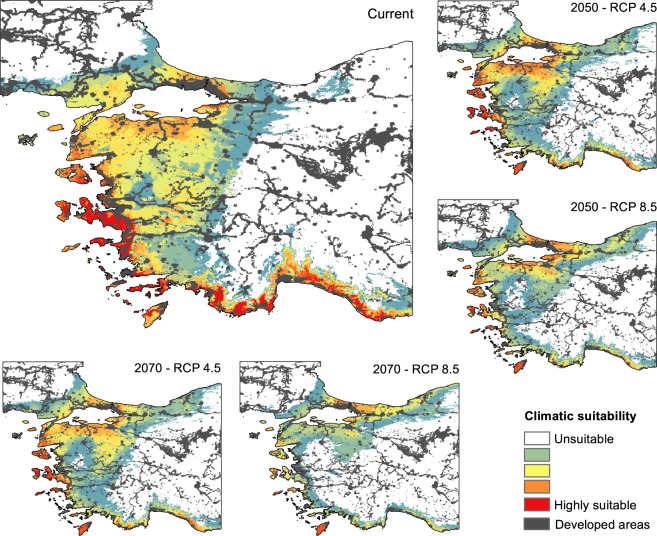
Predicted climatically suitable areas for the Xanthina clade for current and future, 2050 and 2070, with two RCP scenarios. Suitability maps are average calculations of the four GCM models for each time period and RCP scenario. Grey colors show areas with human footprint greater than 50.

**Figure 6:**
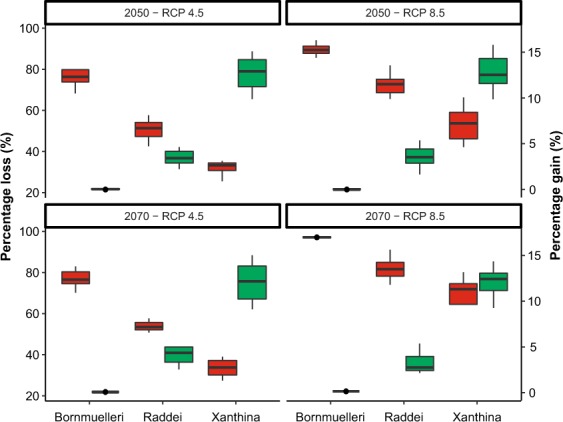
Boxplot of the comparison of percentage loss (red) and gain (green) of climatically suitable patches for the three Montivipera clades under climate change scenarios. Boxplots show variation between four GCM climate projection from ensemble models.

The ecological niche models revealed that suitable habitats of mountain vipers, particularly those of the Bornmuelleri and Raddei clades are patchily spread in mountain ranges under current climates. For the Bornmuelleri clade these areas mainly stretched along the Anatolian diagonal and along two mountainous landscape parallel to the Mediterranean Sea, Mount Lebanon and Anti-Lebanon Mountain. Suitable habitats for the Raddei clade were found in the highlands of the Zagros and Alborz mountains within Iran, and in Ararat and Lesser Caucasus in Turkey and Armenia. For the Xanthina clade the predicted suitable habitats were stretched close to coastal lowlands of the Mediterranean Sea in Turkey. The area under the ROC curve (AUC) and true skill statistics (TSS) values of the 10-fold replicated split-sample tests showed excellent prediction accuracy, as all models revealed an average AUC > 0.8 (except the SRE model for Bornmuelleri and Xanthina) and an average TSS > 0.6 among replicates (Fig. 2). Among the four algorithms, GBM presented the highest and SRE the lowest model accuracies, both for AUC and TSS. Highest model accuracies were generally obtained for the Raddei clade. The AUC of the consensus models of the Bornmuelleri, Raddei, and Xanthina clades were 0.925, 0.939 and 0.911, respectively, and the according TSS values were 0.786, 0.825 and 0.733. The consensus distributions of all three *Montivipera* clades indicated a continuous pattern of range contraction (reduction in projected AOO) under changing climates during the twenty-first century (from the current to 2050 and to 2070) and for RCP scenarios (from RCP 4.5 to RCP 8.5) (see Figs 3,4,5 for the three species and Table 1). The extent of range shifts was quite consistent and similar under the two dispersal scenarios, i.e. no and unlimited dispersal, hence we focused on the no dispersal range changes hereafter. However, the magnitude and direction of range changes vary by clade and emission scenario, with the Bornmuelleri clade showing the highest and the Xanthina group representing the lowest loss in projected AOO (Fig. 6). As we expected, range contractions increase over time and under the more severe emissions scenarios. Accordingly, the extent of projected AOO would get the least reduction by 2050 and using RCP 4.5 (34.6%, 52.1% and 77.6% for the Xanthina, Raddei and Bornmuelleri clades, respectively), while the highest reduction was found under the severe scenario, RCP 8.5 by 2080 (63.8%, 78.3% and 96.8% for Xanthina, Raddei and Bornmuelleri, respectively; Table 1).

**Table 1:** The mean percentage of the extent of suitability stable habitats (Stbl) and range change (RngChg) of PGMCs under future climate conditions (2050 and 2070 time-slice, RCP scenarios 4.5 and 8.5), predicted using ensemble ecological niche models.

	2050–RCP 4.5		2050–RCP 8.5		2070–RCP 4.5		2070–RCP 8.5	
	Stbl	RngChg	Stbl	RngChg	Stbl	RngChg	Stbl	RngChg
Bornmuelleri	22.5 (9.1)	−77.5 (9.2)	13.5 (3.1)	−86.9 (8.2)	22.9 (6.5)	−77.1 (6)	3.4 (0.8)	−96.6 (1.1)
Raddei	45.6 (5.3)	−48.2 (5.4)	36.1 (7)	−63.9 (7.3)	50.2 (5.1)	−49.8 (5.3)	24.9 (3)	−75.1 (3.2)
Xanthina	73.9 (5.5)	−26.1 (8.6)	58.6 (13)	−41.4 (12)	78.2 (11)	−21.8 (5.5)	41.1 (15)	−58.9 (11)

Values in the parenthesis refer to the standard deviation between four GCM models. Negative values indicate range contractions.

The assessment of extinction risks revealed that the Bornmuelleri clade is most at risk (i.e. Threat 2) as it is projected to lose 97% of its AOO by 2070 under RCP 8.5. For the Raddei and Xanthina clades the highest risk was projected to be categorized as Threat 3 (78% and 64% range loss, respectively; Fig. 6) by 2070 under RCP 8.5. Regarding range gain statistics, we also found that the Bornmuelleri clade exhibited a complete absence of gaining newly suitable habitats (percent gain = 0) under any future climate scenario, whilst the Xanthina group was projected to gain the highest proportion of newly suitable habitats (10–11%; Fig. 6).

**Figure 7:**
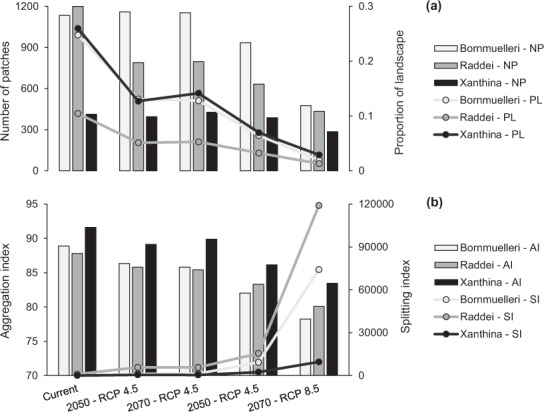
Projected changes in the landscape metrics number of patch (NP), proportion of the landscape (PL), aggregation index (AI) and splitting index (SI) of climatically suitable patches for Montivipera species in eastern Mediterranean Basin.

The results from the landscape analysis showed a decreasing trend in the extent and number of patches projected to remain climatically suitable for all *Montivipera* clades. While, the decreasing trend in the number of patches for the Xanthina clade was low, the decreasing trend of the average patch size was striking for this group (Fig. 7a). Also, both the patch isolation (i.e. aggregation index) and the patch fragmentation (i.e. splitting index) showed increasing trends from current to projected future climates (Fig. 7a). This was particularly the case for the mountain-dwelling clades Bornmuelleri and Raddei, which in turn were reasonably consistent with their projected altitudinal upward shifts (Table 2).

**Table 2:** Projected changes in average altitude of patches climatically suitable for *Montivipera* groups from the current to the future climate scenarios.

	Current	2050–RCP 4.5	2050–RCP 8.5	2070–RCP 4.5	2070–RCP 8.5
Bornmuelleri	1746	2045 (299)	2072 (326)	2256 (510)	2583 (837)
Raddei	2237	2438 (201)	2622 (385)	2430 (193)	2890 (653)
Xanthina	447	412 (−35)	321 (−126)	425 (−22)	323 (−124)

Values in parenthesis show altitudinal shift, in meter, under climate change scenarios compared to the current time.

## Discussion

Most previous studies have found that the impact of climate change on biodiversity range dynamics emerges through projected range shifts and/or range shrinkage^6,12,22,47^. While range shift can be attributed to poleward latitudinal range displacements, as is a case for species inhabiting extensive geographic areas, the range shrinkage often occurs in range-restricted species, often inhabiting mountains, and results from upward altitudinal range contractions^16,48^. The result of projections under GCMs and emissions scenarios in our study revealed a consistent response of mountain vipers in terms of range contractions, originating from an altitudinal shift to higher elevations (Tables 1 and 2; Fig. 2–6). However, the three clades showed varying degrees of range loss under climate change. The Bornmuelleri clade was projected to lose almost all suitable habitats under the RCP 8.5 by 2070. From a geographic and geomorphologic point of view, the remaining suitable habitats in this clade, which are projected to shift towards a warm Mediterranean climate regime, consist of mountainous landscapes close to flat coastal areas.

With respect to the significant climate shifts projected by 2100^49,50^, the Mediterranean Basin will become an important hotspot for biodiversity conservation^51^. Our results are thus consistent with recent updates on projected species habitat losses due to global warming in the Mediterranean and Middle East^7,52,53^. Nevertheless, the effects of climate change on species range shifts in the eastern Mediterranean has not properly been investigated in the literature, and the few existing studies have only focused on very local patterns^7,54^. A key finding of this study is that there is a strong difference in the climate change impacts among the three species clades inhabiting three different eastern Mediterranean sub-regions. Accordingly, our results highlight a significant sub-regional variation in the impacts of climate change on biodiversity, specifically in wide-ranged species such as mountain vipers. Previous studies on climate change projection over the period 1979–2100 have revealed that among the Middle East - North Africa domain the alpine ecosystems of Turkey and Iran are likely to face the largest increase in temperatures, particularly during March-May^49^. This time of the year coincides with the post-hibernation mating activities for *Montivipera* species. Accordingly, apart from projected reductions in the mountain vipers’ suitable habitats and increasing habitat fragmentation, climate changes might also interfere with the species reproductive cycles, and thus their demographic rates.

While there is a proliferation in studies analyzing the projected impact of climate change on biodiversity range shifts^16,22^, bioclimatic niche projections mostly neglect important drivers of ecological integrity^55^, and often fail to address habitat connectivity, despite being an important element for successful conservation of biodiversity under a changing climate^56,57^. Here, by using metrics of patch configuration, we incorporated the concept of landscape integrity to assess the impact of climate change on the capacity for dispersal and long-term resistance of the species. Furthermore, we eliminated areas that are unsuitable because of human land transformation as extracted from a human footprint dataset. This allowed us to incorporate human-induced barriers into our landscape integrity analysis and increased the realism in projections to the landscape scale. Our approach demonstrated that significant proportions of climatically suitable habitats of mountain vipers are both inaccessible and/or highly fragmented due to the dominance of humanized developments. The decline in both the extent and number of suitable habitat patches was consistent among all species, with the Raddei clade showing a lower decrease in the number of patches compared to the Xanthina and Bornmuelleri clades, although its suitable habitat range was highly reduced (Fig. 7a). We thus conclude that the currently suitable habitat patches for the latter two groups will likely become too small for supporting viable populations in the future, as they will lose many of these small marginal patches. For the former, the currently suitable habitat represents core patches that will still remain suitable in the future, albeit losing extent.

The configuration-isolation indices (i.e. AI and SI; Fig. 7a,b) revealed that mountain vipers will be facing an extreme rate of degradation in habitat quality due to increased isolation and fragmentation under projected future climates. The decrease in the aggregation index is caused by increased distance among subpopulations which is the primary factor of reduced connectivity^58^. The impact of climate change on connectivity is more deteriorating for range-restricted species, such as *Montivipera* species, as their population dynamics is suffering from their strongly-limited dispersal behaviors^55,59^. In addition to isolation, mountain vipers’ suitable ranges are increasingly subdivided into smaller patches under projected future warming, which in turn will increase their vulnerability to climate change. Generally, species’ vulnerability to environmental stress is thought to depend on three main elements: exposure, sensitivity and adaptive capacity^60^. In the case of mountain vipers, the high magnitude of range loss due to climate change (i.e. exposure^21,61^) and human-induced barriers to connectivity impose high levels of vulnerability. Mountain vipers are ecological specialists distributed among isolated populations, thus exhibiting a patchy distribution. Their reduced population sizes and life histories exhibiting long generations limit their adaptive capacity to changing climates (i.e. sensitivity^10,62^), which additionally intensify their vulnerability to changing climates. Finally, ongoing trailing-edge fragmentation, limited dispersal capability, lack of gene flow between disjunct populations and low effective population size due to declining population trends all lead to likely low genetic diversity within populations (i.e. adaptive capacity^22,63,64^) which adds again to the vulnerability of mountain vipers to climate change. Given the reduction in the projected AOO of the PGMCs we then assigned them to Threat levels (see Methods for more details on Threat levels). Of the three PGMCs, Bornmuelleri is at highest risk to go extinct and would be classified as IUCN Threat 2, but very close to critically threatened (Threat 1) supported by projections of all GCM models. Overall, the uncertainty in range loss values due to the different climate change models that we considered was very low (SD = 3.8–9.2% for Raddei, SD = 1.1–9.7% for Bornmuelleri, SD = 3.2–12.26% for Xanthina) leading to all three groups being assigned into only one threat level. There are additional drivers that could intensify the risk of species extinctions. For example, catastrophic events such as climate extremes, pathogens, large fires or spread of invasive organisms, although occurring at a low or unpredictable frequency, may impose severe consequences and irreversible effects^65^. Increasing human pressure and land use change, which have been intensifying particularly in the eastern Mediterranean Basin and the Middle East^53^ are important threats to the connectivity of the *Montivipera*’s remnant populations. In addition to habitat destruction, land use degradation hampers the effective efficiency of mountain vipers to track climate change and hence increases the vulnerability of these species. The species have to disperse to areas that are becoming newly suitable under climate change. If populations cannot disperse at the velocity of environment shifts, the species may only persist in remaining refugia^66,67^. The capability to disperse across landscapes may be further reduced by human developments that dissect suitable habitats into isolated patches, thus reducing habitat connectivity and increasing barriers to dispersal^68^. Altogether, the likely fact that most species will not be able to balance their distributional dynamics with the velocity of environmental changes (e.g. future climate change), highlights the necessity of considering ‘assisted migration’ as an adaptive conservation strategy^67^. Moreover, regarding the recent decline in natural populations of some of the *Montivipera* species (e.g. *M. latifii* and *M. wagneri*), *ex situ* conservation, for example captive breeding and introduction-reintroduction programs, could be considered as a strategy to assist the long-term persistence of the species in areas that are highly sensitive to climate changes.

Although EOO and AOO are globally accepted as surrogates of species’ geographic range to assess extinction risk, their application to assign species to risk categories have remained controversial^65^ due to issues of spatial scale and availability of data^33,36^. The finer the scale at which the distribution of species is mapped, the larger will be the area within which the taxon is recorded to be absent, and the more likely the assessed extinction risks will be higher. Conversely, coarse-resolution mapping of species distributions fails to map unoccupied areas, resulting in lower assessed extinction risks. Moreover, mapping species at coarse resolution increases the likelihood of neglecting environmental heterogeneity and hidden local microclimatic refugia, particularly in mountain areas^5,69^. The assessment of climate change impacts and extinction risks is thus scale-dependent, with finer spatial scales usually being more representative of a species’ biology than coarse resolution assessments. In our study, the ensemble models were developed at a relatively fine grid size (ca. 1 km^2^) through which we included local topographic heterogeneity effects and cryptic climate refugia into the range estimations and risk assessments.

In this study, we performed ENM analyses at a clade level above the species rank rather than at the species level due to the sparse availability of data on species distributions. This procedure assumes that niche conservatism holds for recently diversified species in mountain vipers, which is likely the case in most species that have evolved mostly from allopatric speciation. This speciation mode tends to retain ancestral ecological traits (e.g. ecological niche)^70^. In fact, niche conservatism in climatic tolerance might have limited the geographic range expansion of mountain vipers and may have resulted in very similar species-specific ecological niches in this genus. However, we admit that our approach of phylogenetically lumping occurrence points may have introduced some bias. First, our approach expands geographic range size of the species by assigning them to clades. Given the positive correlation between species’ range size and niche breadth^71^, and given that aggregations may smooth over smaller lower-ranked taxonomic units^72^ this method may inflate niche breadth and overestimate the range of suitable habitats for species-specific local populations. The resulting habitat suitability maps may thus be rather optimistic. In reality, there will likely be fewer suitable patches available to any species than what is modelled at the clade level, particularly in the absence of unlimited dispersal. Another key assumption of ENMs is that a species is in equilibrium with the climate^67,73^, meaning that the observed distribution represents the total of suitable habitats of the species. Regarding the evolutionary history of *Montivipera*, high degrees of new-endemism, isolated distribution to mountain and limited dispersal abilities^45,74^ might have eradicated the species from some areas that once were suitable. This could mean that *Montivipera* species might be isolated from otherwise suitable habitats, thus violating to some degree the equilibrium assumption. If true, this would underestimate the true width of suitable habitats, and thus, counteract the overestimation imposed by aggregating species to clades. We believe that both effects are considerably small given the very clear and specific niche identified by our models.

Combining results of population demographic models (e.g. population viability analysis) with distribution models allows for a more accurate prediction of a species extinction risk due to a more detailed representation of the demographic processes^75^. Due to the lack of demographic data, we were not able to construct more dynamic models (e.g. mechanistic models), and we focused on projections of AOO as a proxy of effective habitat loss. Our results thus need to be considered with caution. However, our model-derived estimates of AOO could be useful to design conservation areas^76^, spatial priority-setting implications and conservation planning of the highly endangered mountain vipers.

## Methods

### Study area and Montivipera occurrences

The study area encompasses parts of the eastern Mediterranean Basin, extends to the Near and Middle East and contains two biodiversity hotspots, the Mediterranean Basin and the Caucasus, which are both rich in endemics from a faunal and also from a floristic viewpoint. From a geomorphologic viewpoint, the study area is predominantly mountainous with a small coastal fringe in the west to an increasing alpine system stretching towards the east, starting from the Taurus Mountain in Turkey and ending with the Lesser Caucasus in Armenia and with the Alborz and Zagros Mountains in Iran. Although this region mainly receives its humidity and rainfall from the Mediterranean Sea, long dry seasons have caused the formation of a heterogeneous mixture of sclerophyllous cover types spanning from alpine steppe grasslands to mixed and deciduous wood-forest belts dominated by oaks, junipers, and pines.

Species occurrences used as input to ENM were obtained from field sampling between 2011 and 2016 (particularly for species of the Raddei complex, n = 84) and from other herpetologists’ field studies (n = 62), extractions from existing databases (i.e. Herp-Net and GBIF, n = 16), and from scientific publications (n = 10). In order to avoid effects from spatial autocorrelation of the presence points due to different protocols of field sampling^77^ we randomly sub-selected one point per 10 km radius buffer using the *sp* package^78^ in R v. 3.4.3^79^. In summary, we retained 152 records ranging from 7 for *M. b. albizona* to 37 for *M. xanthina*.

Mountain vipers have a highly isolated distribution with incompletely sampled data. Hence, the insufficient data of the species occurrence is too sparse for performing ENMs at the species level. By lumping occurrence points of the closely related sister-clades we developed niche models above species level^80^. To do so, using a recently constructed phylogenetic tree of the genus^45^ (see Fig. 1), which subdivides the genus into geographically well-separated clades, we divided the current Mediterranean distribution of the genus into three phylogeographic *Montivipera* clades (PGMC). These PGMCs represent three different bioregions including coastal habitats (western Turkey), nearshore mountain habitats (southern Turkey, Lebanon, and Syria) and inland mountainous habitats (northern and western Iran and southern Caucasus), which encompass the core area of the distribution of the three phylogenetic clades Xanthina, Bornmuelleri, and Raddei, respectively (Fig. 1a,b). The ENM analyses were performed such that all occurrences were pooled by PGMCs for further modelling in order to have sufficient observations to build models, obtaining 37, 80 and 35 occurrence points for Bornmuelleri, Raddei and Xanthina clades, respectively. However, we removed the Taurus occurrences of the Xanthina sub-clade because of its more mountainous distribution compared to the rest of the Xanthina sub-clade (see Fig. 1c).

### Ecological niche modelling approach and climate change

To track the impact of climate change on the distribution of *Montivipera* clades we conducted ENM based on climatic variables at a ~1 km resolution derived from WorldClim^81^. We only used climate variables rather than other vegetation-derived predictors in the ENM analysis in order to assess changes of the climatic suitability and to avoid circular reasoning, as vegetation properties themselves depend on climate. In order to sample the whole study area for background contrast, we allocated 10,000 pseudo-absences spatially at random. We then extracted the values of all 19 bioclim variables for presence and pseudo-absence points and calculated the degree of multicollinearity among them based on the variance inflation factor (VIF). To do so, we used the ‘*usdm*’ package^82^ and set a VIF value of 6 and a correlation threshold of 0.75, as recommended by Guisan, *et al*.^83^. As a result, we retained 6 bioclim variables for modelling, including annual mean temperature (bio1), temperature seasonality (bio4), maximum temperature in the warmest month (bio5), annual precipitation (bio12), precipitation of driest month (bio14) and precipitation seasonality (bio15).

To assess the habitat suitability of the species, we fitted four ENM methods, including generalized linear models (GLM), generalized boosting models (GBM), surface range envelop (SRE, or commonly called as BIOCLIM), and maximum entropy (MaxEnt) and combined them into a final ensemble model using *biomod2* package^84^. Using the four methods allowed us to combine simple regression (GLM) and envelop (BIOCLIM) models and complex machine learning (MaxEnt, GBM) models^85^ and thus to assess uncertainties arising from the choice of methods. For GLM we used simple and quadratic terms and stepwise selection procedure based on Akaike Information Criteria (AIC) to fit the best model. GBM was set to allow up to 2500 trees, and we set the learning rate to 0.001 and the bag fraction to 0.5. To conduct SRE the species envelop was set up with a quantile 0.05% to avoid the most extreme values. For MaxEnt all feature types (linear, quadratic, product, threshold and hinge) were allowed and a maximum iteration of 200 was used. For all models we weighted presences and pseudo-absences inverse-proportional so as to give them equal weights and the models’ predictive performance was evaluated by means of a repeated (10×) split-sample test (75% training, 25% evaluation) for which we calculated the AUC and the TSS. The final ensemble model was obtained by weighted-averaging the individual models proportionally to their AUC scores^84^.

The future habitat suitability of the clades was estimated by projecting fitted models to four different global circulation models (GCM), including CCSM4^86^, CNRM-CM5^87^, HadGEM2-ES^88^ and MIROC5^89^ for two time periods of 2050 (average for 2041–2060) and 2070 (average for 2061–2080). Using four different GCMs allowed us to assess the uncertainty from selecting GCMs as these differ clearly among regions^90^. We included a moderate and an extreme greenhouse gas emission scenario in our analyses^91^. For each GCM, we considered two representative concentration pathways (RCPs), namely RCP 4.5 and RCP 8.5. To obtain a final ENM projection, we averaged the ensemble rasters arising from projecting the ENMs to the four GCMs for both scenarios and for the two time periods. Data for bioclim variables based on the four GCMs scenarios and time periods were downloaded from WorldClim at a ~1 km resolution. For each PGMC, we therefore implemented 640 projections (4 ENM algorithms × 10 replications × 4 GCMs × 2 RCPs × 2 time-periods).

To track changes in suitable habitats we calculated the percentage of cells that gained or lost climatic suitability for the four GCM models, for the two RCPs, and for 2050 and 2070, compared to the current area of suitable habitat for each PGMC. To identify suitable cells from unsuitable ones, we first converted continuous ensemble models into binary presence/absence maps based on the minimum habitat suitability score at occurrence points per PGMC as a threshold. Using this approach, we calculated the Minimal Predicted Area (MPA), a minimum area where the species is projected to exist^92^, for establishing the potential AOO for all clades. For this step, we assumed that all current occurrence points represent suitable habitat condition for the species and we did not remove marginal occurrences as “unsuitable”. Then, based on the number of pixels gained or lost under future climatic scenarios we calculated the respective changes in projected AOO (i.e. range shifts) of the groups. We analysed shifts in suitable habitats by employing two different dispersal scenarios, i.e. unlimited dispersal and no dispersal as two extremes (for more details on dispersal scenarios see reference number^93^).

To obtain an accurate prediction of suitable habitats and to avoid inaccessible areas being identified as suitable for mountain vipers, such as developed areas, we masked these human transformed areas from climatically suitable habitats. Accordingly, we also incorporated the human landscape context into suitability projections. We identified developed areas based on the human footprint model developed and implemented by Sanderson *et al*. (2002)^94^. This layer incorporates data on population density and the presence of infrastructures including road networks, land transformation and human access with pixel values ranging from zero (undeveloped) to 100 (cities and highly developed areas). For our study, we assigned areas with values greater than 50 as developed areas and masked them from the predicted habitat suitability maps. We admit that these humanized areas will expand in the future. Yet, due to the lack of available data for future conditions, the mask was applied unchanged to the future climate scenarios. However, urban growth and change in developed areas might not severely alter the results given the large spatial scale of our analyses.

Next, we assessed the severity of future extinction risks of the clades by categorizing them into four threat levels using IUCN Red List Criteria following Maiorano, *et al*.^53^. Threat levels are assigned based on the projected AOO losses, with 1: 100%, 2: <100% and > = 80%, 3: <80% and > = 50%, and 4: <50% and > = 30%. Finally, we assessed the spatial configuration of the suitable habitats of the clades by borrowing landscape ecology metrics that express facilitation or suppression of dispersal and long-term persistence of the clades. To do so, we calculated the number of patches (NP), the proportion of the landscape (PL), the aggregation index (AI), and the splitting index (SI) of suitable habitat patches under current and projected future climates. AI is a metric of spatial adjacency and demonstrates the frequency at which different pairs of patches appear side-by-side in a landscape^95^. The more clumped and compact the patches, the higher are the values of AI. SI measures the degree of fragmentation and can be interpreted as the number of patches with a constant size when the landscape is subdivided into S patches, where S is the value of the splitting index^95^. SI increases as the landscape is increasingly subdivided into smaller patches. All metrics were calculated using the ‘*SDMTools*’ R package. A more detailed description of the metrics is available in McGarigal, *et al*.^95^.

## Data Availability

The datasets generated and analysed during the current study are available from the corresponding author on reasonable request.

## References

[1] Alroy J (2015). Current extinction rates of reptiles and amphibians. Proc Natl Acad Sci USA.

[2] Araújo MB, Thuiller W, Pearson RG (2006). Climate warming and the decline of amphibians and reptiles in Europe. J. Biogeogr..

[3] Pounds JA (2006). Widespread amphibian extinctions from epidemic disease driven by global warming. Nature.

[4] Urban MC (2015). Accelerating extinction risk from climate change. Science.

[5] Randin CF (2009). Climate change and plant distribution: local models predict high‐elevation persistence. Global Change Biol..

[6] VanDerWal J (2013). Focus on poleward shifts in species’ distribution underestimates the fingerprint of climate change. Nat Clim Change.

[7] Yousefi M (2015). Upward Altitudinal Shifts in Habitat Suitability of Mountain Vipers since the Last Glacial Maximum. PLoS ONE.

[8] Dullinger S (2012). Extinction debt of high-mountain plants under twenty-first-century climate change. Nat Clim Chang.

[9] Fordham DA (2016). Predicting and mitigating future biodiversity loss using long-term ecological proxies. Nat Clim Chang.

[10] Pearson RG (2014). Life history and spatial traits predict extinction risk due to climate change. Nat Clim Change.

[11] D’Amen M, Zimmermann NE, Pearman PB (2013). Conservation of phylogeographic lineages under climate change. Global Ecol. Biogeogr..

[12] González-Orozco CE (2016). Phylogenetic approaches reveal biodiversity threats under climate change. Nat Clim Chang.

[13] Pio DV (2014). Climate change effects on animal and plant phylogenetic diversity in southern Africa. Global Change Biol..

[14] Thuiller W (2011). Consequences of climate change on the tree of life in Europe. Nature.

[15] Chen I-C, Hill JK, Ohlemüller R, Roy DB, Thomas CD (2011). Rapid range shifts of species associated with high levels of climate warming. Science.

[16] Garcia RA, Cabeza M, Rahbek C, Araújo MB (2014). Multiple dimensions of climate change and their implications for biodiversity. Science.

[17] Wiens JJ (2010). Niche conservatism as an emerging principle in ecology and conservation biology. Ecol. Lett..

[18] Wüest RO, Antonelli A, Zimmermann NE, Linder HP (2015). Available climate regimes drive niche diversification during range expansion. Am Nat.

[19] Lenoir J (2010). Going against the flow: potential mechanisms for unexpected downslope range shifts in a warming climate. Ecography.

[20] Rumpf SB (2018). Range dynamics of mountain plants decrease with elevation. Proc. Natl. Acad. Sci. USA.

[21] Thomas CD (2004). Extinction risk from climate change. Nature.

[22] Parmesan C (2006). Ecological and evolutionary responses to recent climate change. Annu. Rev. Ecol. Evol. Syst..

[23] Foden WB (2013). Identifying the world’s most climate change vulnerable species: a systematic trait-based assessment of all birds, amphibians and corals. PLoS ONE.

[24] IUCN. IUCN Red List Categories and Criteria: Version 3.1. Second edition. Gland, Switzerland and Cambridge, UK: IUCN. iv + 32pp. (2012).

[25] Boitani L (2008). Distribution of medium-to large-sized African mammals based on habitat suitability models. Biodivers. Conserv..

[26] Syfert MM (2014). Using species distribution models to inform IUCN Red List assessments. Biol. Conserv..

[27] Jiménez-Alfaro B, Draper D, Nogués-Bravo D (2012). Modeling the potential area of occupancy at fine resolution may reduce uncertainty in species range estimates. Biol. Conserv..

[28] Araújo MB, Whittaker RJ, Ladle RJ, Erhard M (2005). Reducing uncertainty in projections of extinction risk from climate change. Global Ecol. Biogeogr..

[29] Thuiller W (2004). Uncertainty in predictions of extinction risk. Nature.

[30] Engler R, Hordijk W, Guisan A (2012). The MIGCLIM R package – seamless integration of dispersal constraints into projections of species distribution models. Ecography.

[31] González‐Suárez M, Lucas PM, Revilla E (2012). Biases in comparative analyses of extinction risk: mind the gap. J. Anim. Ecol..

[32] Scheffers BR, Joppa LN, Pimm SL, Laurance WF (2012). What we know and don’t know about Earth’s missing biodiversity. Trends Ecol. Evol..

[33] Akçakaya HR (2000). Making consistent IUCN classifications under uncertainty. Conserv. Biol..

[34] Boakes EH (2010). Distorted views of biodiversity: spatial and temporal bias in species occurrence data. PLoS Biol..

[35] Joseph LN, Possingham HP (2008). Grid-based monitoring methods for detecting population declines: sensitivity to spatial scale and consequences of scale correction. Biol. Conserv..

[36] Gaston KJ, Fuller RA (2009). The sizes of species’ geographic ranges. J. Appl. Ecol..

[37] Stümpel, N. & Joger, U. In *Animal Biodiversity in the Middle East. Proceedings of the First Middle Eastern Biodiversity Congress*, *Aqaba, Jordan*. 20–23.

[38] Abrantes, F. *et al*. In *Mediterranean Climate: from Past to the Future* (ed Piero Lionello) 1–86 (Elsevier Inc. 2010).

[39] Molina-Venegas R, Aparicio A, Lavergne S, Arroyo J (2016). Climatic and topographical correlates of plant palaeo-and neoendemism in a Mediterranean biodiversity hotspot. Ann. Bot..

[40] Tzedakis P (2007). Seven ambiguities in the Mediterranean palaeoenvironmental narrative. Quat Sci Rev.

[41] Popov, S. V. *et al*. Lithological-Paleogeographic maps of Paratethys-10 maps Late Eocene to Pliocene. (2004).

[42] Ruiz C, Jordal BH, Serrano J (2012). Diversification of subgenus Calathus (Coleoptera: Carabidae) in the Mediterranean region–glacial refugia and taxon pulses. J. Biogeogr..

[43] Verdú M, Pausas JG (2013). Syndrome-driven diversification in a Mediterranean ecosystem. Evolution.

[44] Sindaco, R., Venchi, A., Carpaneto, G. M. & Bologna, M. A. The reptiles of Anatolia: a checklist and zoogeographical analysis. *Biogeographia* **21** (2000).

[45] Stümpel N, Rajabizadeh M, Avcı A, Wüster W, Joger U (2016). Phylogeny and diversification of mountain vipers (Montivipera, Nilson et al., 2001) triggered by multiple Plio–Pleistocene refugia and high-mountain topography in the Near and Middle East. Mol. Phylogen. Evol..

[46] Behrooz R (2015). Habitat modeling and conservation of the endemic Latifi’s Viper (*Montivipera latifii*) in Lar National Park, Northern Iran. Herpetol Conserv Biol.

[47] Walther G-R (2002). Ecological responses to recent climate change. Nature.

[48] Dirnböck T, Essl F, Rabitsch W (2011). Disproportional risk for habitat loss of high‐altitude endemic species under climate change. Global Change Biol..

[49] Bucchignani E, Mercogliano P, Panitz H-J, Montesarchio M (2018). Climate change projections for the Middle East–North Africa domain with COSMO-CLM at different spatial resolutions. Advances in Climate Change Research.

[50] Giorgi F, Lionello P (2008). Climate change projections for the Mediterranean region. Global Planet. Change.

[51] Giorgi, F. Climate change hot‐spots. *Geophys. Res. Lett*. **33** (2006).

[52] Barredo JI, Caudullo G, Dosio A (2016). Mediterranean habitat loss under future climate conditions: Assessing impacts on the Natura 2000 protected area network. Appl Geogr.

[53] Maiorano L (2011). The future of terrestrial mammals in the Mediterranean basin under climate change. Philos Trans R Soc B.

[54] Al-Qaddi N, Vessella F, Stephan J, Al-Eisawi D, Schirone B (2017). Current and future suitability areas of kermes oak (Quercus coccifera L.) in the Levant under climate change. Reg Environ Chang.

[55] Wasserman T, Cushman S, Shirk A, Landguth E, Littell J (2012). Simulating the effects of climate change on population connectivity of American marten (Martes americana) in the northern Rocky Mountains, USA. Landscape Ecol..

[56] Heller NE, Zavaleta ES (2009). Biodiversity management in the face of climate change: a review of 22 years of recommendations. Biol. Conserv..

[57] Krosby M, Tewksbury J, Haddad NM, Hoekstra J (2010). Ecological connectivity for a changing climate. Conserv. Biol..

[58] Hodgson JA, Thomas CD, Wintle BA, Moilanen A (2009). Climate change, connectivity and conservation decision making: back to basics. J. Appl. Ecol..

[59] Velo‐Antón G, Parra J, Parra‐Olea G, Zamudio K (2013). Tracking climate change in a dispersal‐limited species: reduced spatial and genetic connectivity in a montane salamander. Mol. Ecol..

[60] Small-Lorenz SL, Culp LA, Ryder TB, Will TC, Marra PP (2013). A blind spot in climate change vulnerability assessments. Nature Climate Change.

[61] Ofori BY, Stow AJ, Baumgartner JB, Beaumont LJ (2017). Influence of adaptive capacity on the outcome of climate change vulnerability assessment. Sci. Rep..

[62] Cabrelli AL, Stow AJ, Hughes L (2014). A framework for assessing the vulnerability of species to climate change: a case study of the Australian elapid snakes. Biodivers. Conserv..

[63] Aitken, S. N. & Whitlock, M. C. Assisted gene flow to facilitate local adaptation to climate change. *Annu Rev Ecol Evol Syst* **44** (2013).

[64] Hoffmann AA, Sgro CM (2011). Climate change and evolutionary adaptation. Nature.

[65] Mace GM (2008). Quantification of extinction risk: IUCN’s system for classifying threatened species. Conserv. Biol..

[66] Hargreaves AL, Eckert CG (2014). Evolution of dispersal and mating systems along geographic gradients: implications for shifting ranges. Funct. Ecol..

[67] Wiens JJ, Stralberg D, Jongsomjit D, Howell CA, Snyder MA (2009). Niches, models, and climate change: assessing the assumptions and uncertainties. Proceedings of the National Academy of Sciences.

[68] Cushman, S. A. *et al*. In *Key Topics in Conservation Biology 2*. (eds Macdonald, D. W. & Willis, K. J.) 384–404 (Wiley-Blackwell, 2013).

[69] Thuiller W, Lavorel S, Araújo MB, Sykes MT, Prentice IC (2005). Climate change threats to plant diversity in Europe. Proc Natl Acad Sci USA.

[70] Wiens JJ, Graham CH (2005). Niche conservatism: integrating evolution, ecology, and conservation biology. Annu. Rev. Ecol. Evol. Syst..

[71] Moore TE, Bagchi R, Aiello-Lammens ME, Schlichting CD (2018). Spatial autocorrelation inflates niche breadth–range size relationships. Global Ecol. Biogeogr..

[72] Pearman PB, D’amen M, Graham CH, Thuiller W, Zimmermann NE (2010). Within‐taxon niche structure: niche conservatism, divergence and predicted effects of climate change. Ecography.

[73] Guisan A, Zimmermann NE (2000). Predictive habitat distribution models in ecology. Ecol. Model..

[74] Behrooz R (2018). Conservation Below the Species Level: Suitable Evolutionarily Significant Units among Mountain Vipers (the Montivipera raddei complex) in Iran. J. Hered..

[75] Fordham DA (2012). Plant extinction risk under climate change: are forecast range shifts alone a good indicator of species vulnerability to global warming?. Global Change Biol..

[76] Brambilla M (2009). GIS-models work well, but are not enough: Habitat preferences of Lanius collurio at multiple levels and conservation implications. Biol. Conserv..

[77] Hijmans RJ, Phillips S, Leathwick J, Elith J, Hijmans MRJ (2017). Package ‘dismo’. Circles.

[78] Bivand, R. S., Pebesma, E. J., Gómez-Rubio, V. & Pebesma, E. J. *Applied spatial data analysis with R*. Vol. 747248717 (Springer 2008).

[79] R Core Team. *R: A language and environment for statistical computing*., (R Foundation for Statistical Computing, Vienna, Austria, 2016).

[80] Smith, A. B., Godsoe, W., Rodríguez-Sánchez, F., Wang, H.-H. & Warren, D. Niche Estimation Above and Below the Species Level. *Trends Ecol. Evol*. (2018). 10.1016/j.tree.2018.10.012 30497791

[81] Hijmans RJ, Cameron SE, Parra JL, Jones PG, Jarvis A (2005). Very high resolution interpolated climate surfaces for global land areas. Int J Climatol.

[82] Naimi B (2015). usdm: Uncertainty analysis for species distribution models. R package version.

[83] Guisan, A., Thuiller, W. & Zimmermann, N. E. *Habitat Suitability and Distribution Models: With Applications in R*. (Cambridge University Press, 2017).

[84] Thuiller W, Lafourcade B, Engler R, Araújo MB (2009). BIOMOD–a platform for ensemble forecasting of species distributions. Ecography.

[85] Merow C (2014). What do we gain from simplicity versus complexity in species distribution models?. Ecography.

[86] Gent PR (2011). The community climate system model version 4. J. Clim..

[87] Voldoire A (2013). The CNRM-CM5. 1 global climate model: description and basic evaluation. Clim Dyn.

[88] Jones C (2011). The HadGEM2-ES implementation of CMIP5 centennial simulations. Geoscientific Model Development.

[89] Watanabe M (2010). Improved climate simulation by MIROC5: mean states, variability, and climate sensitivity. J. Clim..

[90] Flato G (2013). Evaluation of Climate Models. In: Climate Change 2013: The Physical Science Basis. Contribution of Working Group I to the Fifth Assessment Report of the Intergovernmental Panel on Climate Change. . Climate Change.

[91] Van Vuuren DP (2011). The representative concentration pathways: an overview. Clim. Change.

[92] Engler R, Guisan A, Rechsteiner L (2004). An improved approach for predicting the distribution of rare and endangered species from occurrence and pseudo‐absence data. J. Appl. Ecol..

[93] Engler R (2009). Predicting future distributions of mountain plants under climate change: does dispersal capacity matter?. Ecography.

[94] Sanderson EW (2002). The human footprint and the last of the wild: the human footprint is a global map of human influence on the land surface, which suggests that human beings are stewards of nature, whether we like it or not. Bioscience.

[95] McGarigal, K., Cushman, S. A., Neel, M. C. & Ene, E. FRAGSTATS: spatial pattern analysis program for categorical maps. *University of Massachusetts, Amherst*. (2002).

